# Synthesis, Antiinflammatory and HIV-1 Integrase Inhibitory Activities of 1,2-Bis[5-thiazolyl]ethane-1,2-dione Derivatives

**DOI:** 10.4103/0250-474X.56021

**Published:** 2009

**Authors:** P. X. Franklin, S. Yerande, H. M. Thakar, G. S. Inamdar, R. S. Giri, H. Padh, V. Sudarsanam, Kamala K. Vasu

**Affiliations:** Medicinal Chemistry Department, B. V. Patel Pharmaceutical Education and Research Development Centre, Thaltej, Ahmedabad-380 054, India; 1 Biotechnology Department, B. V. Patel Pharmaceutical Education and Research Development Centre, Thaltej, Ahmedabad-380 054, India

**Keywords:** N,N-diethyl benzamidine, N,N-diethyl phenylacetamidine, 1,4-dibromo butane-2,3-dione, benzoylisothiocyanate

## Abstract

Based on principles of pharmacophore delineation and drug designing, compounds containing diketofunctionallity namely 1,2-bis[5-thiazolyl]ethane-1,2-diones were designed and synthesized as antiinflammatory agents. The compounds were evaluated in carrageenan-induced rat-paw edema method. G-3, G-6, G-17, G-20, G-23, G-22, L-708 and 906 showed good antiinflammatory activity. In addition as diketo functionality containing compounds are reported to have HIV-1 integrase inhibitory property, and these compounds contains diketo functionality, so these compounds were screened in assay for HIV-1 integrase inhibition. Few compounds showed weak HIV-1 integrase Inhibitory activity.

Rheumatoid arthritis is a chronic inflammatory disorder which affects 1-2% of the total world population, commonly leads to significant disability and a consequent reduction in quality of life[1]. The available antirheumatic drugs include NSAIDs, drugs acting on cytokines and disease modifying antirheumatic drugs (DMARDs)[2]. Treatment using NSAIDs, only provide symptomatic relief. TNF-α is a cytokine and inhibitors of this cytokine eg. Etanercept, Infliximab and IL-1 inhibitor anakira, which act on cytokines, have demonstrated clinical efficacy in the treatment of rheumatoid arthritis. But treatments with these agents are costly and require parenteral administration[3]. Even though DMARDs (e.g., methotrexate, cyclosporine) inhibit the disease progression, the adverse effects of these drugs are very unpalatable[4,5]. The unmet medical need calls for drug discovery programs for discovery of drugs which inhibit the disease progression, which are safe and with oral bioavailability.

Our earlier drug discovery program for the treatment of inflammatory disorders found the template of substituted 2-amino thiazol-5-yl-oxo (fig. 1A) ideal for designing drugs for inflammatory disorders[6]. There are several literature reports which highlight the fact that when 2 “minimum structural features necessary for activity” are incorporated into one single molecule the activity increases many folds[7,8]. Novel bisquinoline analogue (I) with chloroquine-like features was found to inhibit both chloroquine sensitive and resistant *P. falciparum* malarial parasites (IC_50_ -17 nM in resistant strains as compared to chloroquine which was 540 nM)[9]. Another example is the bis-(difluro methylene phosphonate) analogue which was more active as a protein tyrosine phosphatase 1B inhibitor as compared to the monophosphonate[10]. Literature nowadays is replete with instances of such idea. Applying this strategy to the template we synthesized a series 1,2-bis[5-thiazolyl]ethane-1,1-diones as antiinflammatory agents.

**Fig 1:**
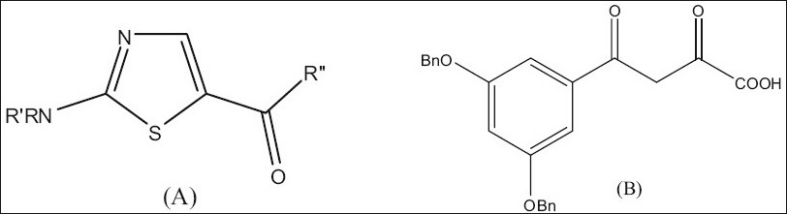
Substituted 2-amino thiazol-5-yl-oxo and diketoaryl motifs. Templates of A. substituted 2-amino thiazol-5-yl-oxo and B. Diketoaryl motifs

Integrase (IN) has emerged as an attractive target, because it is necessary for stable infection and homologous enzymes are lacking in the human host[11]. The function of IN is to catalyze integration of proviral DNA, resulting from the reverse transcription of viral RNA, into the host genome. This is achieved in a stepwise fashion by endonucleolytic processing of proviral DNA within a cytoplasmic preintegration complex (termed 3' P-processing or “3'-P”), followed by translocation of the complex into the nuclear compartment where integration of 3'-processed proviral DNA into host DNA occurs in a “strand transfer” (ST) reaction. Although numerous agents potently inhibit 3C'-P and ST in extracellular assays that employ recombinant IN and viral long terminal repeat oligonucleotide sequences, often such inhibitors lack inhibitory potency when assayed using fully assembled preintegration complexes or fail to show antiviral effects against HIV infected cells. Recently, however, a class of IN inhibitors has emerged, typified by an aryl-diketo motif. Variations of this structural theme have been utilized by Shionogi and Merck pharmaceutical companies to prepare inhibitors (for example, L-708,906) which potently block integration in extracellular assays and exhibit good antiviral effects against HIV-infected cells. (fig. 1B)[11,12]. The presence of diketo pharmacophore in the synthesized compounds made us to screen these compounds in a HIV-1 integrase inhibitory assay.

## MATERIALS AND METHODS

Phenyl isothiocyanate was procured from Lancaster. Methyl isothiocyanate was gift sample from Hitesh Chemicals and Drugs Pvt Ltd, Hyderabad, India. Benzoylisothiocyanate, methoxycarbonyl isothiocyanate, ethoxy carbonyl isothiocyanate, N,N-diethyl actamidine, N,N-diethyl benzamidine, N,N-diethylphenyl acetamidine and 1,4-dibromo butane 2,3-dione were prepared in our lab following the reported procedures[13–17]. Carrageenin was procured from Spectrochem India limited. HIV-1 integrase assay was done at Department of Pharmaceutical Sciences, School of Pharmacy, University of Southern California, Los Angeles, USA[18]. The reported protocol was followed for carrageenin-induced rat paw edema method[19] for evaluation of antiinflammatory activity. All compounds were screened in a group of 6 animals at dose of 100 mg/kg body weight of the animal. The experimental protocol was approved by the institutional animal ethics committee, constituted as per CPCSEA, Government of India.

### General method of preparation of intermediates with amidines, Scheme 1:

**Scheme 1:**
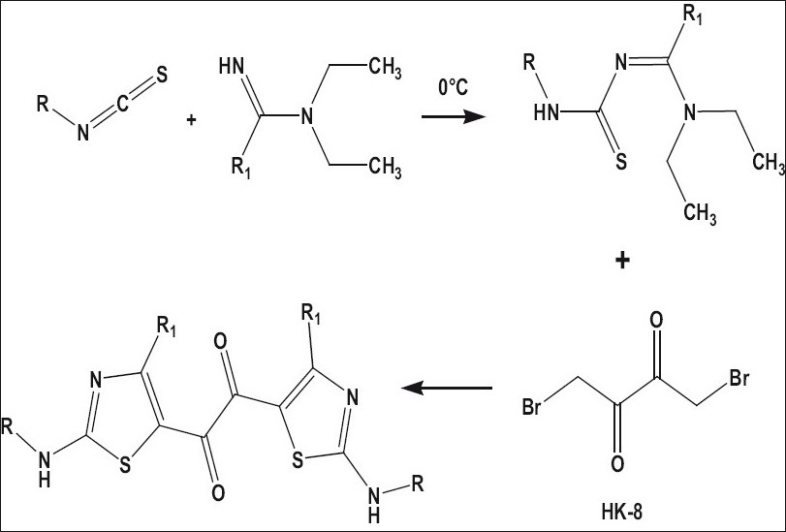
General method of preparation of intermediates with amidines. General method of synthesis of 1,2-bis[2-(alkyllamino)-4-alkyl-1,3-thiazol-5-yl]ethane-1,2-ones

The scheme reported by Rajappa *et al*. was utilized for the synthesis of the target molecules[20–23]. Various isothiocyanates were reacted with N,N-diethyl acetamidine, N,N-diethyl benzamidine, N,N-diethyl phenylacetamidine to give the adducts. Some other adducts were synthesized by reacting benzoylisothiocyanate with corresponding secondary amines. The final compounds were synthesized by the reaction of 1,4-dibromo butane-2,3-dione, with the adducts. For preparation of adducts, the isothiocyanates were stirred with amidines in toluene at 0°. The solid that separated was filtered washed with cold toluene and dried.

### General method of preparation of intermediates with secondary amines, Scheme 2:

**Scheme 2:**
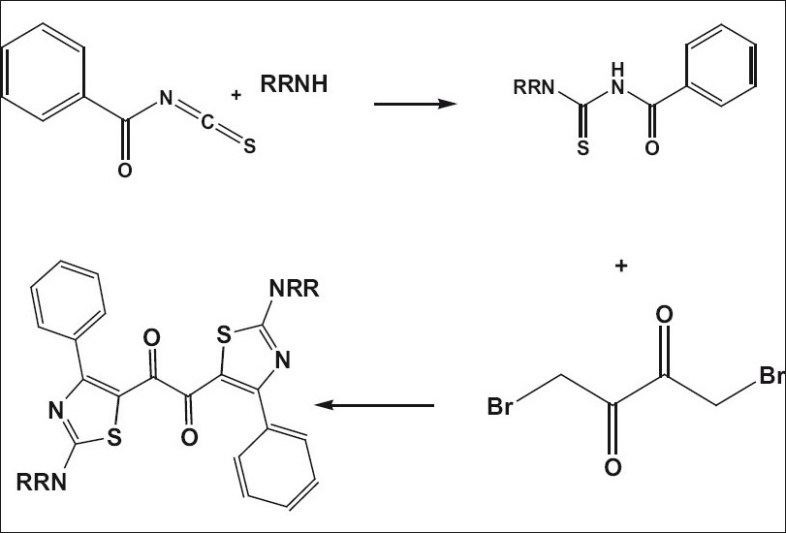
General method of preparation of intermediates with secondary amines. General method of synthesis of 1,2-bis[2-(dialkyllamino)-4-phenyl-1,3-thiazol-5-yl]ethane-1,2-ones

The solution of benzoyl chloride and ammonium thiocyanate in acetone in a round bottom flask attached with a reflux condenser, were refluxed for 15 min. To this corresponding secondary amine was added and refluxing was continued for another 2 h. After cooling the reaction mixture was poured into excess ice cold water. The solid that separated was filtered and dried to obtain the intermediates.

### Preparation of 1,4-dibromo butane-2,3-dione[24]:

1,4-Dibromo butane-2,3-dione was prepared by modification of a reported method. In a three necked round bottomed flask, 10 ml CHCl_3_ was taken. In separating funnels diacetyl (20 g, 0.23 mol) and bromine (37.2 g, 0.46 mol) were taken in 50 and 30 ml CHCl_3,_ respectively. Both the solutions were added drop wise simultaneously into the flask by keeping a little excess of bromine in the flask throughout the addition. Disappearance of bromine colour was observed during the reaction. The orange colour solution was kept at 0° for 8 h. The solid that separated was filtered. The filtrate was washed with NaHCO_3_ solution dried and concentrated to get II crop 1,4-dibromo butane-2,3-dione. Yield 71%, mp 170°.

### General procedure for synthesis of the final compounds:

Two equivalents of adduct and one equivalent of 1,4-dibromo butane-2,3-dione was dissolved in acetonitrile and stirred for 24 h, the solid that separated was filtered, washed with NaHCO_3_ and recrystalised from DMF/acetonitrile mixture. The physical data of all the synthesized compounds is presented in Table 1.

**TABLE 1:** PHYSICAL DATA OF SYNTHESIZED COMPOUNDS

Code	Molecular Formula	Formula Weight	M. P. (°)	LC-MS (M+1)	IR (KBr cm^−1^)	NMR (δ ppm)
G-1	C_12_H_14_N_4_O_2_S_2_	310.39	*	311	3212, 2634, 1592, 1465, 1395, 1276, 1237, 1023, 809	(d_6_DMSO) 11.25(2H, s, 2NH), 7.09-7.63(10H m, aromatic), 2.26(12H, s, 4CH_3_)
G-2	C_22_H_18_N_4_O_2_S_2_	434.53	*	435	3197, 2939, 1602, 1558, 1451, 1368, 1329, 1227, 1023, 799	
G-3	C_22_H_16_C_l2_N_4_O_2_S_2_	503.42	*	504	3173, 2930, 1611, 1560, 1494, 1467, 1425, 1369, 1337, 1232, 1211, 1096, 1012, 801	
G-4	C_14_H_14_N_4_O_6_S_2_	398.41	*	399	3173, 2954, 1733, 1633, 1558, 1500, 1371, 1212, 815, 760	
G-5	C_16_H_18_N_4_O_6_S_2_	426.46	*	427	3163, 1732, 1634, 1553, 1478, 1324, 1298, 1215, 815,	
G-6	C_34_H_22_N_4_O_4_S_2_	614.49	234-6	615	3314, 1675, 1644, 1522, 1454, 1409, 1330, 1298, 1223, 1099, 926, 816, 703, 659,	
G-7	C_22_H_18_N_4_O_2_S_2_	434.53	250-2	435	3184, 2989, 1682, 1658, 1441, 1358, 1339, 1220, 1023, 760	
G-8	C_32_H_22_N_4_O_2_S_2_	558.67	>265	559	1622, 1600, 1557, 1508, 1451, 1431, 1431, 1335, 1279, 1211, 939, 802, 771, 755,	(d_6_DMSO) 11.06(2H, s, 2NH), 7.10-7.95(20H m, aromatic)
G-9	C_32_H_20_C_l2_N_4_O_2_S_2_	627.56	*	628	1621, 1515, 1497, 1453, 1335, 1277, 1235, 1073, 794, 770,	
G-10	C_24_H_18_N_4_O_6_S_2_	522.55	*	523	3345, 1753, 1659, 1528, 1513, 1465, 1336, 1327, 1214, 1198, 754,	
G-11	C_26_H_22_N_4_O_6_S_2_	550.60	252-5	551	3335, 1745, 1639, 1531, 1504, 1455, 1326, 1307, 1224, 1195, 804	
G-12	C_34_H_26_N_4_O_2_S_2_	586.72	*	587	1609, 1555, 1539, 1476, 1450, 1361, 1312, 1156, 985, 804, 776	
G-13	C_34_H_24_C_l2_N_4_O_2_S_2_	655.61	261-3	656	3176, 3035, 1603, 1552, 1494, 1458, 1408, 1355, 1230, 1089, 828, 716	
G-14	C_26_H_22_N_4_O_6_S_2_	550.60	*	551	3178, 3036, 2954, 1738, 1645, 1558, 1480, 1339, 1208, 1072, 814, 765,	
G-15	C_28_H_26_N_4_O_6_S_2_	578.66	*	579	3329, 1742, 1632, 1540, 1465, 1197, 1058, 799,	
G-16	C_36_H_26_N_4_O_4_S_2_	642.74	*	643	3315, 3029, 1682, 1632, 1542, 1469, 1406, 1356, 1295, 1215, 1093, 898, 806, 690	
G-17	C_30_H_30_N_4_O_2_S_2_	542.71	235 d	543	2939, 1663, 1606, 1552, 1465, 1446, 1281, 940, 774	
G-18	C_28_H_26_N_4_O_2_S_2_	546.66	155-7	547	1646, 1609, 1579, 1486, 1456, 1354, 1293, 1180, 929, 702,	
G-19	C_30_H_32_N_6_O_2_S_2_	572.74	220 d	573	3421, 2944, 1733, 1697, 1683, 1661, 1638, 1532, 1300, 1205, 1076, 950, 780	
G-20	C_32_H_36_N_6_O_2_S_2_	600.79	198-200	601	3173, 2983, 2837, 1689, 1601, 1580, 1539, 1489, 1464, 1430, 1385, 1365, 1314, 1284, 1259, 1230, 1138, 1021, 722, 660	
G-21	C_40_H_36_N_6_O_2_S_2_	696.88	189-90	697	1663, 1619, 1533, 1498, 1456, 1350, 1350, 1326, 1294, 1227, 1026, 1001, 936, 770	
G-22	C_42_H_40_N_6_O_2_S_2_	724.93	178-80	725	1688, 1600, 1532, 1264, 1228, 1153, 1117, 939, 865, 700	

* Indicates that these compounds have melting point higher than 300°

## RESULTS AND DISCUSSION

Many enzymes exist as dimers in their active form. This may be the reason that the dimerised pharmacophores shows better activity by binding through the appropriate epitopes in a facile manner at the dimeric binding site of the macromolecule. Here we dimerised the pharmacophore showing antiinflammatory activity developed in our laboratory (Tables 2 and 3). Most of the dimeric compounds showed better antiinflammatory activity than their parent monomer type compound. Few compounds like G-6, G-7, G-8, G-12, G-16, G-17, G-18, G-19, G-20 and G-21 gave protection by more than 50% in the carrageenan rat paw model. The compounds with a bulky and lipophilic group at the 2 and 2' position showed excellent antiinflammatory activity. However, representative examples of these classes of compounds did not have any effect on TNF-α- release from human peripheral blood mononuclear cells (G-6, G-17, G-19) and one compound weakly inhibited cPLA_2_(G-6).

**TABLE 2:** ANTIINFLAMMATORY ACTIVITY OF 1,2-BIS[2-(ALkYL/ARYLAMINO)-4-ALkYL/ARYL-1,3-THIAZOL-5-YL]ETHANE-1,2-ONES 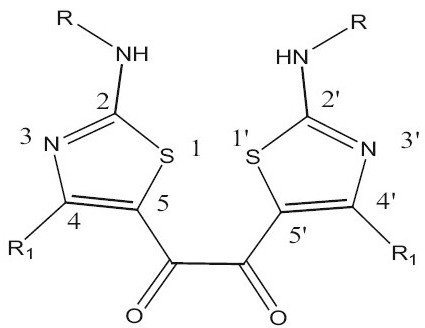

CODE	R	R1	% PROTECTION Dose 100 mg/kg b.w
G-1	CH_3_	CH_3_	14±7
G-2	C_6_H_5_	CH_3_	23±8
G-3	p-Cl-C_6_H_4_	CH_3_	73±0.3
G-4	CH_3_OCO	CH_3_	40±2
G-5	C_2_H_5_OCO	CH_3_	33±0.9
G-6	C_6_H_5_CO	C_6_H_5_	74±0.5
G-7	C_6_H_5_	C_6_H_5_	70±0.7
G-8	p-Cl-C_6_H_4_	C_6_H_5_	66±1
G-9	CH_3_OCO	C_6_H_5_	26±3
G-10	C_2_H_5_OCO	C_6_H_5_	34±4
G-11	C_6_H_5_	C_6_H_5_CH_2_	23±2
G-12	p-Cl-C_6_H_4_	C_6_H_5_CH_2_	72±1
G-13	CH_3_OCO	C_6_H_5_CH_2_	35±2.5
G-14	C_2_H_5_OCO	C_6_H_5_CH_2_	38±2
G-15	C_6_H_5_CO	C_6_H_5_CH_2_	47±2.3

*Ibuprofen was taken as positive control which 65 % inhibition at 100 mg/kg

**TABLE 3:** ANTIINFLAMMATORY ACTIVITY OF 1,2-BIS[2-(DIALkYLLAMINO)-4-HETERYL-1,3-THIAZOL-5-YL]ETHANE-1,2-ONES 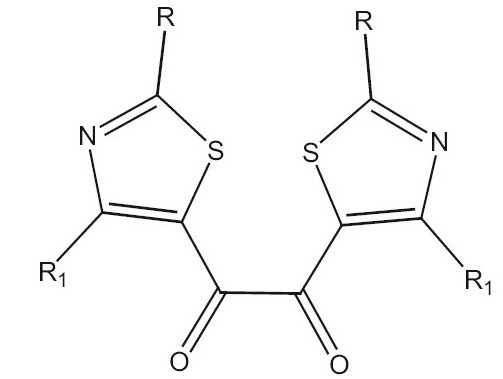

CODE	R	R1	% PROTECTION
G-16	piperidinyl	C_6_H_5_	65±0.8
G-17	4-morpholinyl	C_6_H_5_	81±0.5
G-18	N-methylpiperazinyl	C_6_H_5_	60±0.5
G-19	N-ethylpiperazinyl	C_6_H_5_	72±0.5
G-20	N-phenylpiperazinyl	C_6_H_5_	55±4
G-21	N-benzylpiperazinyl	C_6_H_5_	85±0.3

In the integrase assay few compounds showed weak activity (Table 4). The pharmacophore of the reported compounds contains hetero aryl ring connected by a linker containing diketo functionality. The weak activity of our compounds may be due to the fact that in the reported potent HIV-1 integrase inhibitors, besides the diketo moiety an additional carboxylic acid moiety is present and this feature may also be necessary for good antiintegrase activity. In conclusion the good antiinflammatory activity shown by G3, G6, G7 and G12 seem to indicate the utility of this framework as a lead with a potential to optimize to a developable drug-like candidate to treat inflammatory diseases.

**TABLE 4:** INHIBITORY POTENCIES AS MEASURED IN AN EXTRACELLULAR HIV-1 INTEGRASE ASSAY

Compounds	3'P (μm)	ST (μm)
G-3	132	397
G-6	>500	277
G-17	>500	109
G-20	122	87
G-23	276	276
G-22	287	>500
L-708,906	>1000	0.48

No other compounds showed any activity in concentration range 500 μm
